# Deep Learning and Transformer Approaches for UAV-Based Wildfire Detection and Segmentation

**DOI:** 10.3390/s22051977

**Published:** 2022-03-03

**Authors:** Rafik Ghali, Moulay A. Akhloufi, Wided Souidene Mseddi

**Affiliations:** 1 Perception, Robotics and Intelligent Machines Research Group (PRIME), Department of Computer Science, Université de Moncton, Moncton, NB E1A 3E9, Canada; rafik.ghali@ept.rnu.tn; 2 SERCOM Laboratory, Ecole Polytechnique de Tunisie, Université de Carthage, BP 743, La Marsa 2078, Tunisia; wided.souidene@ept.rnu.tn

**Keywords:** wildfire detection, fire classification, fire segmentation, vision transformers, UAV, aerial images

## Abstract

Wildfires are a worldwide natural disaster causing important economic damages and loss of lives. Experts predict that wildfires will increase in the coming years mainly due to climate change. Early detection and prediction of fire spread can help reduce affected areas and improve firefighting. Numerous systems were developed to detect fire. Recently, Unmanned Aerial Vehicles were employed to tackle this problem due to their high flexibility, their low-cost, and their ability to cover wide areas during the day or night. However, they are still limited by challenging problems such as small fire size, background complexity, and image degradation. To deal with the aforementioned limitations, we adapted and optimized Deep Learning methods to detect wildfire at an early stage. A novel deep ensemble learning method, which combines EfficientNet-B5 and DenseNet-201 models, is proposed to identify and classify wildfire using aerial images. In addition, two vision transformers (TransUNet and TransFire) and a deep convolutional model (EfficientSeg) were employed to segment wildfire regions and determine the precise fire regions. The obtained results are promising and show the efficiency of using Deep Learning and vision transformers for wildfire classification and segmentation. The proposed model for wildfire classification obtained an accuracy of 85.12% and outperformed many state-of-the-art works. It proved its ability in classifying wildfire even small fire areas. The best semantic segmentation models achieved an F1-score of 99.9% for TransUNet architecture and 99.82% for TransFire architecture superior to recent published models. More specifically, we demonstrated the ability of these models to extract the finer details of wildfire using aerial images. They can further overcome current model limitations, such as background complexity and small wildfire areas.

## 1. Introduction

Forest fire accidents are one of the most dangerous risks due to their frightening loss statistics. The fires cause human, financial, and environmental losses, including the death of animals and the destruction of wood, houses, and million acres of land worldwide. In 2021, forest fires have occurred in several countries such as the European Union countries, the US (United States), central and southern Africa, the Arabian Gulf, and South and North America [1]. They affect 350 million to 450 million hectares every year [2]. In the western US alone, the frequency of wildfires and the total area burned increased by 400% and 600%, respectively, in the last decade [3]. In addition, approximately 8000 wildfires affected 2.5 million hectares each year in Canada [4].

Generally, wildfires are detected using various sensors such as gas, smoke, temperature, and flame detectors. Nevertheless, these detectors have a variety of limitations such as delayed response and small coverage areas [5]. Fortunately, the advancement of computer vision techniques has made it possible to detect fire using visual features collected with cameras. However, traditional fire detection tools are being replaced by vision-based models that have many advantages such as accuracy, large coverage areas, small probability of errors, and most importantly the ability to work with existing camera surveillance systems. Through the years, researchers have proposed many innovative techniques based on computer vision in order to build accurate fire detection systems [6,7,8,9].

In recent years, Unmanned Aerial Vehicles (UAV) or drone systems were deployed in various tasks such as traffic monitoring [10], precision agriculture [11], disaster monitoring [12], smart cities [13], cover mapping [14], and object detection [15]. They are also very practical and well developed for wildfire fighting and detection. UAV-based systems help with precise fire management and provide real-time information to limit damage from fires thanks to their low cost and ability to cover large areas whether during the day or night for a long duration [16,17]. The integration of UAVs with visual and/or infrared sensors help in finding potential fires at daytime and nighttime [18]. Furthermore, fire detection and segmentation showed impressive progress thanks to the use of deep learning (DL) techniques. DL-based fire detection methods are used to detect the color of wildfire and its geometrical features such as angle, shape, height, and width. Their results are used as inputs to the fire propagation models. Thanks to the promising performances of DL approaches in wildfire classification and segmentation [19], researchers are increasingly investigating this family of methods. The existing methods use input images captured by traditional visual sensors to localize wildfire and to detect the precise shape of fire; they achieved high results [20,21,22]. However, it is not yet clear that these methods will perform well in detecting and segmenting forest fire using UAV images, especially in the presence of various challenges such as small object size, background complexity, and image degradation.

To address these problems, we present in this paper a novel deep ensemble learning method to detect and classify wildfire using aerial images. This method employs EfficientNet-B5 [23] and DenseNet-201 [24] models as a backbone for extracting forest fire features. In addition, we employed a deep model (EfficientSeg [25]) and two vision transformers (TransUNet [26] and TransFire) in segmenting wildfire pixels and detecting the precise shape of fire on aerial images. Then, the proposed wildfire classification method was compared to deep convolutional models (MobileNetV3-Large -Small [27], DenseNet-169 [24], EfficientNet-B1-5 [23], Xception [28,29], and InceptionV3 [29]), which showed excellent results for object classification. TransUNet, TransFire, and EfficientSeg are also evaluated with U-Net [28].

More specifically, three main contributions were reported in this paper. First, a novel DL method was proposed to detect and classify wildfire using aerial images in order to improve detection and segmentation of wildland fires. Second, vision transformers were adopted for UAV wildfire segmentation to segment fire pixels and identify the precise shape of the fire. Third, the efficiencies of CNN methods and vision transformers are demonstrated in extracting the finer details of fire using aerial images and overcoming the problems of background complexity and small fire areas.

The remainder of the paper is organized as follows: Section 2 presents recent DL approaches for UAV-based fire detection and segmentation. Section 3 describes the methods and materials used in this paper. In Section 4, experimental results and discussion are presented. Finally, Section 5 concludes the paper.

## 2. Related Works

DL approaches are employed for fire detection and segmentation using aerial images. They proved their ability to detect and segment wildfires [6,20]. They can be grouped into three categories: DL approaches for UAV-based fire classification, DL approaches for UAV-based fire detection, and DL approaches for UAV-based fire segmentation.

### 2.1. Fire Classification Using Deep Learning Approaches for UAV Images

Convolutional Neural Networks (CNNs) are the most popular AI models for images classification tasks. They extract feature maps from input images and then predict their correct classes (two classes in our case: Fire and Non-Fire). Three main types of layers, which are convolutional layers, pooling layers, and fully connected layers, are employed to build a classical CNN architecture:

- Convolution layers are a set of filters designed to extract basic and complex features such as edges, corners, texture, colors, shapes, and objects from the input images. Then, activation functions are used to add the non-linearity transformation. It helps CNN to learn complex features in the input data. Various activation functions were employed, such as Rectified Linear Unit (ReLU) function [30], Leaky ReLU (LReLU) function [31], parametric ReLU (PReLU) function [32], etc.
- Pooling layers reduce the size of each feature map resulting from the convolutional layers. The most used pooling methods are average pooling and max pooling.
- The fully connected layer is fed by the final flattened pooling or convolutional layers’ output, and the class scores for the objects present in the input image are computed.

CNNs showed good results for object classification and recognition [33]. Motivated by their great success, researchers presented numerous CNN-based contributions for fire detection and classification using aerial images in the literature, and these are summarized in Table 1.

Chen et al. [34,39] proposed two CNNs to detect wildfire in aerial images. The first CNN contains nine layers [39]. It consists of a convolutional layer with Sigmoid function, max-pooling layer, ReLU activations, Fully connected layer, and Softmax classifier. Using 950 images collected with a six-rotor drone (DJI S900) equipped with a SONYA7 camera, the experimental results showed improvements in accuracy compared to other detection methods [39]. The second includes two CNNs for detecting fire and smoke in aerial images [34]. Each CNN contains 17 layers. The first CNN classifies Fire and Non-Fire, and the second detects the presence of smoke in the input images. Using 2100 aerial images, great performance (accuracy of 86%) was achieved, outperforming the first method and the classical method, which combines LBP (Local Binary Patterns) and SVM [34]. Lee et al. [35] employed five deep CNNs, which included AlexNet [40], GoogLeNet [41], VGG13 [42], a modified GoogLeNet, and a modified VGG13 to detect forest fires in aerial images:

- AlexNet includes eleven layers: five convolutional layers with ReLU activation function, three max-pooling layers, and three fully connected layers;
- VGG13 is a CNN with 13 convolutional layers;
- GoogLeNet contains 22 inception layers, which employ, simultaneously and in parallel, multiple convolutions with various filters and pooling layers;
- Modified VGG13 is a VGG13 model with a number of channels of each convolutional layer and fully connected layers equal to half of that of the original VGG13;
- Modified GoogLeNet is a GoogLeNet model with a number of channels of each convolutional layer and fully connected layer equal to half of that of the original GoogLeNet.

GoogLeNet and the modified GoogLeNet achieved high accuracies thanks to data augmentation techniques (cropping, vertical, and horizontal flip). They showed their ability in detecting wildfires in aerial images [35]. Shamsoshoara et al. [28] proposed a novel method based on the Xception model [43] for wildfire classification. Xception architecture is an extension of the Inceptionv3 model [44] with the modified depth-wise separable convolution, which contains 1×1 convolution followed by a n×n convolution and no intermediate ReLU activations. Using 48,010 images of the FLAME dataset [45] and data augmentation techniques (horizontal flip and rotation), this method achieved an accuracy of 76.23%. Treneska et al. [29] also adopted four deep CNNs, namely InceptionV3, VGG16, VGG19, and ResNet50 [46], to detect wildfire in aerial images. ResNet50 achieved the best accuracy with 88.01%. It outperformed InceptionV3, VGG16, and VGG19 and the recent state-of-the-art model, Xception, using transfer learning techniques and the FLAME dataset as learning data. Srinivas et al. [37] also proposed a novel method, which integrates CNN and Fog computing to detect forest fire using aerial images at an early stage. The proposed CNN consists of six convolutional layers followed by the ReLU activation function and max-pooling layers, three fully connected layers, and a sigmoid classifier that determines the output as Fire or Non-Fire. This method showed a great performance (accuracy of 95.07% and faster response time) and proved its efficiency to detect forest fires. Zhao et al. [36] presented a novel model called “Fire_Net” to extract fire features and classified them as Fire and Non-Fire. Fire_Net is a deep CNN with 15 layers. It consists of eight convolutional layers with ReLU activation functions, four max-pooling layers, three fully connected layers, and a softmax classifier. Using the UAV_Fire dataset [36], Fire_Net achieved an accuracy of 98% and outperformed previous methods. Wu et al. [38] used a pretrained MobileNetv2 [47] model to detect both smoke and fire. MobileNetv2 is an extended version of MobileNetv1 [48], which is a lightweight CNN with depth-wise separable convolutions. It requires small data and reduces the number of parameters of the model and its computational complexity. It employs inverted residuals and linear bottlenecks to improve the performance of MobileNetv1. Using transfer learning and data augmentation strategies, this method achieved an accuracy of 99.3%. It outperformed published state-of-the-art methods such as Fire_Net and AlexNet and proved its suitability in detecting forest fire on aerial monitoring systems [38].

### 2.2. Fire Detection Using Deep Learning Approaches for UAV

Region-based CNNs are used to detect, identify, and localize objects in an image. They determine the detected objects’ locations in the input image using bounding boxes. These techniques are divided into two categories: one-stage detectors and two-stage detectors [49]. One-stage detectors detect and localize objects as a simple regression task in an input image. Two-stage detectors generate the ROI (Region of Interest) in the first step using the region proposal network. Then, the generated region is classified and its bounding box is determined. Region-based CNNs showed excellent accuracy for object detection problems. They are also employed to reveal the best performance in detecting fires on aerial images.

Table 2 presents deep learning methods for UAV-based fire detection. Jiao et al. [50] exploited the one-stage detector, YOLOv3 [51], to detect forest fires. YOLOv3 is the third version of YOLO deep object detectors. It was proposed to improve the detection performance of older versions by making detections at three different scales and using the Darknet-53 model, which contains 53 convolutional layers as a backbone [51]. Testing results revealed great performances and high speed [50]. Jiao et al. [52] also proposed the UAV-FFD (UAV forest fire detection) platform, which employs YOLOv3 to detect smoke and fire by using UAV-acquired images. YOLOv3 showed high performance with reduced computational time (F1-score of 81% at a frame rate of 30 frames per second). It proved its potential in detecting smoke/fire with high precision in real-time UAV applications [52]. Alexandrov et al. [53] adopted two one-stage detectors (SSD [54] and YOLOv2 [55]) and a two-stage detector (Faster R-CNN [56]) to detect wildfires. Using large data of real and simulated images, YOLOv2 showed the best performance compared to Faster R-CNN, SSD, and hand-crafted classical methods. It proved its reliability in detecting smoke at an early stage [53]. Tang et al. [57] also presented a novel application to detect wildfire using 4K images, which have a high resolution of 3840 × 2160 pixels collected by UAS (Unmanned Aerial Systems). A coarse-to-fine strategy was proposed to detect fires that are sparse, small, and irregularly shaped. At first, an ARSB (Adaptive sub-Region Select Block) was employed to select subregions, which contain the objects of interest in 4K input images. Next, these subregions were zoomed to maintain the area bounding box’s size. Then, YOLOv3 was used to detect the objects. Finally, the bounding boxes in the subregions were combined. Using 1400 4K aerial images, this method obtained a mean average precision (mAP) of 67% at an average speed of 7.44 frames per second.

### 2.3. Fire Segmentation Using Deep Learning Approaches for UAV

Image segmentation is very important in computer vision. It determines the exact shape of the objects in the images. With the progress of deep learning models, numerous problems were tackled and a variety of solutions was proposed with good results.

Deep learning models are also used to segment fire pixels and detect the precise shape of smoke and/or flame using aerial images. Table 3 shows deep learning methods for UAV-based fire segmentation. For example, Barmpoutis et al. [58] proposed a 360-degree remote sensing system to segment both fire and smoke using RGB 360-degree images, which were collected from UAV. Two DeepLab V3+ [59] models that are encoder–decoder detectors with ASPP (Atrous Spatial Pyramid Pooling) were applied to identify smoke and flame regions. Then, an adaptive post-validation scheme was employed to reject smoke/flame false-positive regions, especially regions with similar characteristics with smoke and flame. Using 150 360-degree images of urban and forest areas, experiments achieved an F1-score of 94.6% and outperformed recent state-of-the-art methods such as DeepLabV3+. These results showed the robustness of the proposed method in segmenting smoke/fire and reducing the false-positive rate [58]. Similarly to wildfire classification, Shamsoshoara et al. [28] proposed a method based on the encoder–decoder U-Net [60] for wildfire segmentation. Using a dropout strategy and the FLAME dataset, U-Net obtained an F1-score of 87.75% and proved its ability to segment wildfire and identify the precise shapes of flames [28]. Frizzi et al. [61] also proposed a method based on VGG16 to segment both smoke and fire. This method showed good results (accuracy of 93.4% and segmentation time per image of 21.1 s) using data augmentation techniques such as rotation, flip, changing brightness/contrast, crop, and adding noises. It outperformed previous published models and proved its efficiency in detecting and classifying fire/smoke pixels [61].

## 3. Materials and Methods

In this section, we first introduced our proposed methods for wildfire classification and segmentation. Then, we describe the dataset used in training and testing. Finally, we present the evaluation metrics employed in this work.

### 3.1. Proposed Method for Wildfire Classification

To detect and classify fire, we propose a novel method based on deep ensemble learning using EfficientNet-B5 [23] and DenseNet-201 [24] models. EfficientNet models proved their efficiency to reduce the parameters and Floating-Point Operations Per Second using an effective scaling method that employs a compound coefficient to uniformly scale model depth, resolution, and width. EfficientNet-B5 showed excellent accuracy and outperformed state-of-the-art models such as Xception [43], AmoebaNet-A [62], PNASNet [63], ResNeXt-101 [64], InceptionV3 [44], and InceptionV4 [65]. DenseNet (Dense Convolutional Network) connects each layer to all preceding layers to create very diversified feature maps. It has several advantages, including feature reuse, elimination of the vanishing-gradient problem, improved feature propagation, and a reduction in the number of parameters. Using extracted features of all complexity levels, DenseNet shows interesting results in various competitive object recognition benchmark tasks such as ImageNet, SVHN (Street View House Numbers), CIFAR-10, and CIFAR-100 [24].

**Figure 1:**
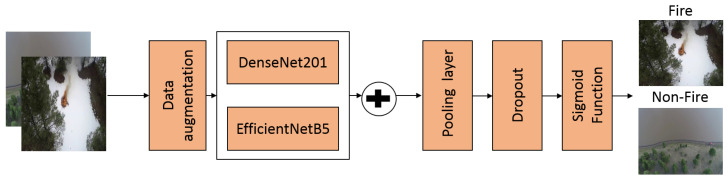
The proposed architecture for wildfire classification.

Figure 1 presents the architecture of the proposed method. First, this method is fed with RGB aerial images. EfficientNet-B5 and DenseNet-201 models were employed as a backbone to extract two feature maps. Next, the feature maps of the two models are concatenated. The concatenated map was then fed an average pooling layer. Then, a dropout of 0.2 was employed to avoid overfitting. Finally, a Sigmoid function was applied to classify the input image into Fire or Non-Fire classes.

### 3.2. Proposed Methods for Wildfire Segmentation

To segment wildfires, we used a CNN model, EfficientSeg [25], and two vision transformers, which are TransUNet [26] and TransFire.

#### 3.2.1. TransUNet

**Figure 2:**
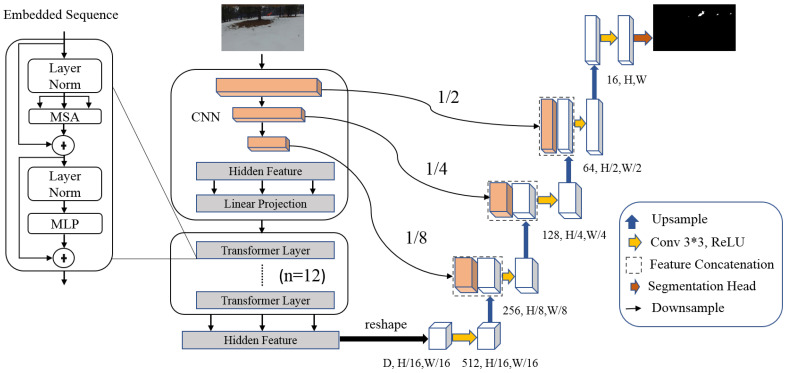
The proposed TransUNet architecture.

TransUNet [26] is a vision transformer based on U-Net architecture. It employs global dependencies between inputs and outputs using self-attention methods. It is an encoder–decoder. The encoder uses a hybrid CNN-transformer architecture consisting of ResNet-50 and pretrained ViT (Vision Transformer) to extract feature maps. It contains MLP (Multi-Layer Perceptron) and MSA (Multihead Self-Attention) blocks. The decoder employs CUP (cascaded up-sampler) blocks to decode the extracted features and outputs the binary segmentation mask. Each CUP includes a 3 × 3 convolutional layer, ReLU activation function, and two upsampling operators. Figure 2 depicts the architecture of TransUNet.

#### 3.2.2. TransFire

TransFire is based on MedT (Medical Transformer) architecture. MedT [66] was proposed in order to segment medical images with no requirement of a large dataset for training. Two concepts, gated position-sensitive axial attention and LoGo (Local-Global) training methodology, were employed to improve segmentation performance. Gated position-sensitive axial attention was used to determine long-range interactions between the input features with high computational efficiency. LoGo training methodology used two branches, which are global branch and local branch, to extract feature maps. The first branch works on the image’s original resolution. It consists of 2 encoders and 2 decoders. The second operates on image patches. It contains 5 encoders and 5 decoders. The input to both of these branches is the feature extracted using a convolutional block, which includes 3 convolutional layers with ReLU activation function and batch normalization.

**Figure 3:**
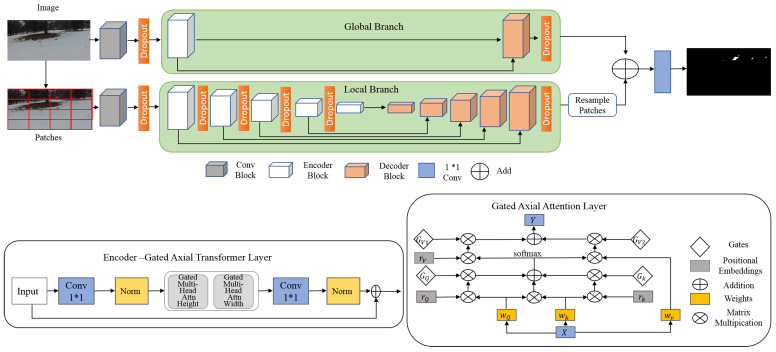
The proposed TransFire architecture.

TransFire is a modified MedT architecture. It includes one encoder and one decoder in the global branch. It also employs a dropout strategy in the local branch (after the fourth first encoders and the last decoder), in the global branch (after the decoder), and in each input of both of these branches. TransFire was developed to overcome the memory problem of MedT and to prevent overfitting. Figure 3 illustrates the architecture of TransFire.

#### 3.2.3. EfficientSeg

EfficientSeg [25] is a semantic segmentation method, which is based on a U-Net structure and uses MobileNetV3 [27] blocks. It showed impressive results and outperformed U-Net in some medical image segmentation tasks [25].

**Figure 4:**
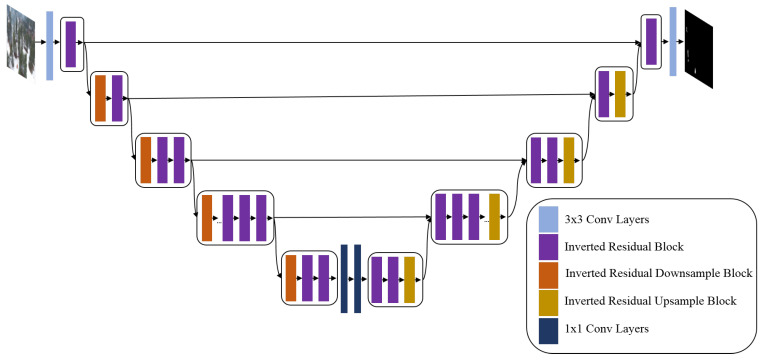
The proposed EfficientSeg architecture.

Figure 4 depicts the architecture of EfficientSeg. It is an encoder–decoder with 4 concatenation shortcuts. It includes five types of blocks, which are MobileNetV3 blocks (Inverted Residual blocks), Downsampling operator, Upsampling operator, and 1 × 1 and 3 × 3 convolutional blocks with ReLU activation function and batch normalization layer.

### 3.3. Dataset

In the area of deep learning, many large datasets are available for researchers to train their models and perform benchmarking by making comparisons with other methods. However, until recently, there was a lack of a UAV dataset for fire detection and segmentation. In this work, we use a public database called FLAME dataset (Fire Luminosity Airborne-based Machine learning Evaluation) [45] to train and evaluate our proposed methods. The FLAME dataset contains aerial images and raw heat-map footage captured by visible spectrum and thermal cameras onboard a drone. It consists of four types of videos, which are a normal spectrum, white-hot, fusion, and green-hot palettes.

**Figure 5:**
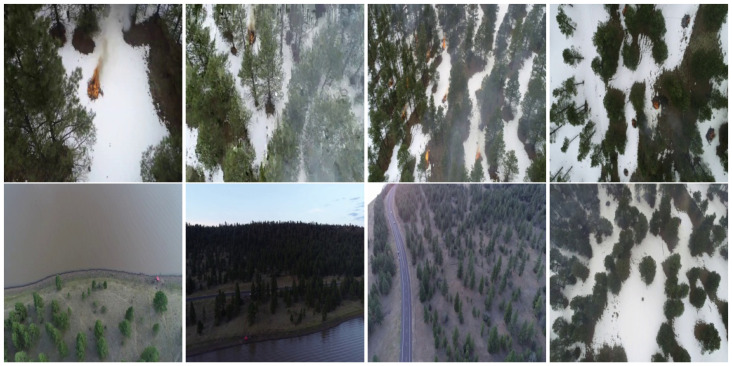
Examples from the FLAME dataset. Top line: Fire images and bottom line: Non-Fire images.

**Figure 6:**
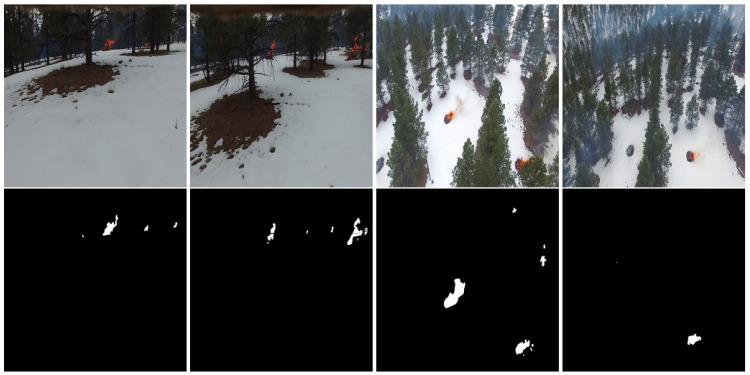
Examples from the FLAME dataset. Top line: RGB images; bottom line: their corresponding binary masks.

In this paper, we focus on RGB aerial images. We used 48,010 RGB images, which are split into 30,155 Fire images and 17,855 Non-Fire images for wildfire classification task. Figure 5 presents some samples of the FLAME dataset for fire classification. On the other hand, we used 2003 RGB images and their corresponding masks for fire segmentation task. Figure 6 illustrates some examples of RGB aerial images and their corresponding binary masks.

### 3.4. Evaluation Metrics

We used F1-score, accuracy, and inference time to evaluate our proposed approaches for fire classification and segmentation:

- F1-score combines precision and recall metrics to determine the ability of the model in detecting wildfire pixels (as shown by Equation (1)):
  (1) $$F1\text{-}\mathrm{score}=\frac{2\times Precision\;\times \;Recall}{Precision+Recall}$$
  (2) $$Precision=\frac{TP}{TP+FP}$$
  (3) $$Recall=\frac{TP}{TP+FN}$$
  where TP is the true positive rate, FP is the false positive rate, and FN is the false negative rate.
- Accuracy is the proportion of correct predictions over the number of total ones, achieved per the proposed model (as given by Equation (4)):
  (4) $$\mathrm{Accuracy}=\frac{TP+TN}{TP+FP+TN+FN}$$
  where TN is the true negative rate, FN is the false negative rate, TP is the true positive rate, and FP is the false positive rate.
- Inference time is the average time of segmentation or classification using our testing images.

## 4. Results and Discussion

For wildfire classification, we used TensorFlow [67] and trained the proposed models on a machine with NVIDIA Geforce RTX 2080Ti GPU. The learning data were split as follows: 31,515 images for training, 7878 images for validation, and 8617 images for testing as presented in Table 4.

We employed categorical cross-entropy loss (*CE*) [68], which measures the probability of the presence of a wildfire in the input image (as shown in Equation (5)):

(5) $$CE=-\sum_{c=1}^{M}z_{b,c}\log\left(p_{b,c}\right)$$

where *M* is the number of classes (in our case two classes (Fire and Non-Fire)), *p* is the predicted probability, and *z* is the binary indicator.

For our experiments, we used input RGB images with 254 × 254 resolution, a batch size of 16, and Adam as an optimizer. We also employed the following data augmentation techniques: rotation, shear, zoom, and shift with random values.

For wildfire segmentation, we developed the proposed methods using Pytorch [69] on an Nvidia V100l GPU. Learning data were divided into three sets: 1401 images for training, 201 images for validation, and 401 images for testing. We employed dice loss [70] to measure the difference between the predicted binary mask and the corresponding input mask (as given by Equation (6)). We also used two data augmentation methods, which are a horizontal flip and a rotation of 15 degrees:

(6) $$DC=1-\frac{2\left|Z⋂W\right|}{\left|Z\right|\;+\;\left|W\right|}$$

where *Z* is the input aerial image, *W* is the predicted image, and ⋂ is the intersection of the input and the predicted images.

The input data are RGB aerial images with a 512 × 512 resolution and their corresponding binary mask. The TransFire Transformer was trained from scratch (no pretraining) using a hybrid CNN-Transformer as a backbone, patch sizes of 16, and a learning rate of 10${}^{-3}$. TransUNet is evaluated using a learning rate of 10${}^{-3}$, patch size of 16, and two backbones that include a pretrained ViT and a hybrid backbone, which includes ResNet50 (R-50) and pretrained ViT. EfficientSeg also was tested from scratch using a learning rate of 10${}^{-1}$.

We analyzed the proposed methods’ performance (accuracy and F1-score) as well as their speed (inference time). In addition, we compared our novel wildfire classification method to state-of-the-art models (Xception [28,29] and InceptionV3 [29]) and deep CNNs (MobileNetV3-Large [27], MobileNetV3-Small [27], DenseNet-169 [24], and EfficientNet-B1-5 [23]), which already showed excellent results for object classification. We also compared the proposed wildfire segmentation methods, including TransUNet, TransFire, and EfficientSeg, to U-Net [28].

### 4.1. Wildfire Classification Results

We trained wildfire classification methods on aerial images collected using the Matrice 200 drone with a Zenmuse X4S camera. Testing data are collected using the Phantom drone with a Phantom camera.

Table 5 reports a comparative analysis of our proposed method and deep CNN methods using the test data. We can observe that our proposed method achieved the best performance (accuracy of 85.12% and F1-score of 84.77%) thanks to scaled and diversified feature maps extracted by EfficientNet-B5 and DenseNet-201 models. It outperformed recent models for object classification (MobileNetV3-Large, MobileNetV3-Small, DensNet-169, and EfficientNet models (EfficientNet-B2, -B3, -B4, and -B5)) and inception models (Xception and InceptionV3). It proved its good ability to detect and classify forest fires on aerial images. However, it needed a high inference time with 0.018 s.

**Figure 7:**
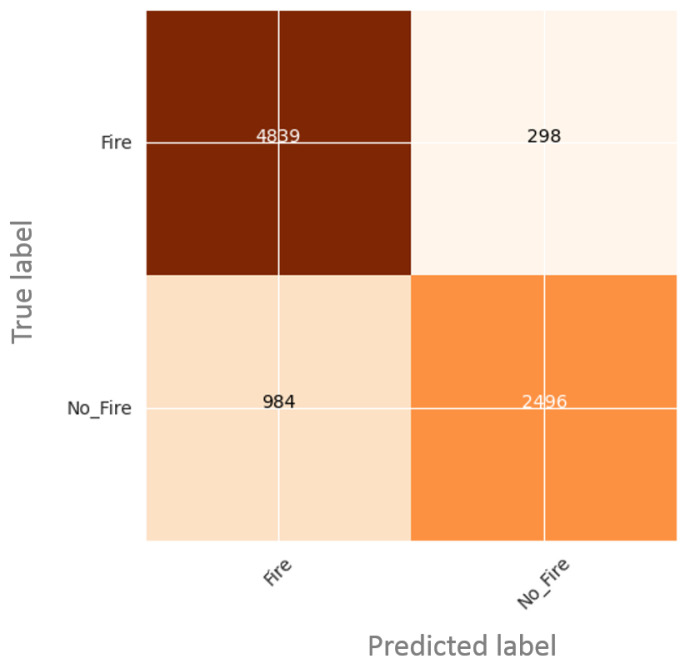
Confusion matrix for fire classification.

Figure 7 presents the confusion matrix on test data. We can see that the rate of true positives (classifying Fire as Fire) and the rate of true negatives (classifying No-Fire as No-Fire) are higher than the rate of the false positives (classifying Fire as No-Fire) and the rate of false negatives (classifying No-Fire as Fire), respectively. Our proposed method showed interesting results in detecting and classifying fires, even for very small fire areas. It proved its efficiency to overcome challenging problems such as uneven object intensity and background complexity.

To conclude, our proposed method revealed the best result based on the trade-off between performance and inference time. It showed an excellent capacity to classify forest fires in aerial images and managed to overcome the problems of small fire areas and background complexity.

### 4.2. Wildfire Segmentation Results

Table 6 illustrates the quantitative results of fire segmentation using the FLAME dataset. We can see that TransUNet, TransFire, and EfficientSeg obtained excellent results and outperformed U-Net used as a baseline model.

Vision Transformers (TransUNet and TransFire) obtained higher performances compared to deep CNN models (EfficientSeg and U-Net) due to their ability to determine long-range interactions within input features and extract the finer details of the input images. TransUNet-R50-ViT achieved the best performance with an accuracy of 99.9% and an F1-score of 99.9% thanks to local and global features extracted using a hybrid backbone, which includes a CNN, R-50, and pretrained ViT Transformer.

**Figure 8:**
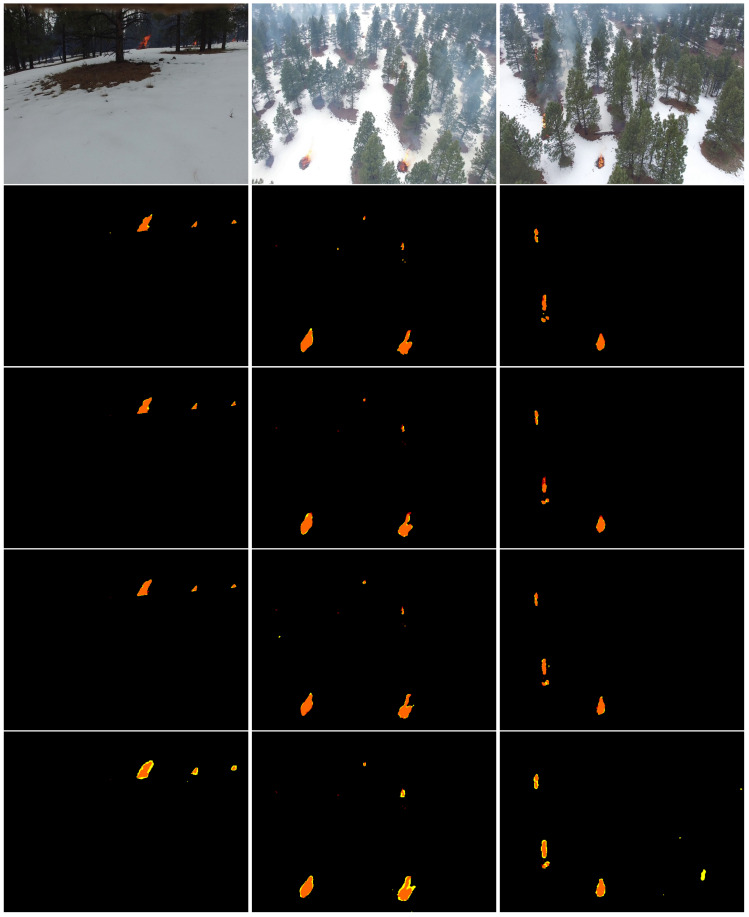
Segmentation results of the proposed models. From top to bottom: RGB aerial images and the results of TransUNet-R50-ViT, TransUNet-ViT, TransFire, and EfficientSeg. Orange represents *TP* (true positives), yellow depicts *FP* (false positives), and red shows *FN* (false negatives).

Figure 8 depicts examples of the segmentation of TransUNet-R50-ViT. We can see that this model accurately detected the finer details of fire and distinguished between wildfire and background. In addition, TransUNet-R50-ViT showed its efficiency in localizing and detecting the precise shape of wildfire, especially with respect to small fire areas on aerial images.

TransUNet-ViT also showed excellent performances (accuracy of 99.86% and F1-score of 99.86%) and high speeds (inference time of 0.4 s) compared to TransFire and EfficientSeg. We can see in Figure 8 that TransUNet with ViT transformer accurately segmented wildfire pixels and detected wildfire regions even for small fire areas.

TransUNet models proved their ability in segmenting wildfire, in detecting the exact shape of fire areas, and in overcoming challenging problems such as small fire areas and background complexity. However, they still depend on a pretrained vision transformer (ViT) on a large dataset.

TransFire also showed a higher accuracy with 99.83% and an F1-score of 99.82% due to high-level information and finer features extracted in the global branch and local branch, respectively. It outperformed EfficientSeg and U-Net. It proved its excellent capacity in segmenting wildfire pixels and detecting the exact fire areas, especially small fire areas as shown in Figure 8. It also segmented forest fire pixels under the presence of smoke.

EfficientSeg also obtained a high accuracy with 99.63% and an F1-score of 99.66% thanks to its extracted finer details. It outperformed U-Net. It showed its efficiency in segmenting fire pixels and in detecting the precise shape of fire areas as depicted in Figure 8. However, It had a higher inference time with 1.38 s compared to vision transformers.

**Figure 9:**
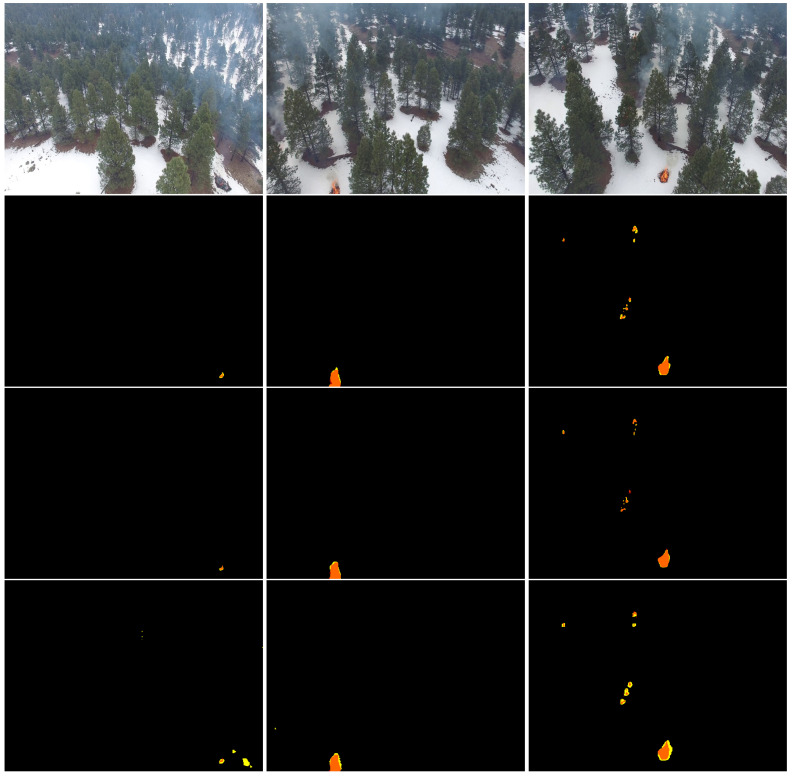
Results of TransFire, TransUNet-R50-ViT, and EfficientSeg. From top to bottom: RGB aerial images and the results of TransFire, TransUNet-R50-ViT, and EfficientSeg. Orange represents *TP*, yellow depicts *FP*, and red shows *FN*. We can see the interesting results of TransFire in determining the precise size of small wildfire areas under the presence of smoke compared to TransUNet and EfficientSeg models.

To conclude, TransUNet, TransFire, and EfficientSeg showed excellent performances. They proved an impressive potential in segmenting wildfire pixels and determining the precise shape of fire. Based on the F1-score, TransFire showed great performance and outperformed deep convolutional models (EfficientSeg and U-Net) and was very close to the performance of vision transformer (TransUNet). In addition, it demonstrated its reliability in detecting and segmenting wildland fires; in particular, it was the best performing in detecting small fire areas under the presence of smoke, as observed in Figure 9.

## 5. Conclusions

In this paper, we address the problem of wildfire classification and segmentation on aerial images using deep learning models. A novel ensemble learning method, which combines EfficientNet-B5 and DenseNet-201 models, was developed to detect and classify wildfires. Using the FLAME dataset, experimental results showed that our proposed method was the most reliable in wildfire classification tasks, presenting a higher performance than recent state-of-the-art models. Furthermore, two vision transformers (TransUNet and TransFire) and a deep CNN (EfficientSeg) are developed to segment wildfires and detect the region of fire areas on aerial images. This is the first proposed approach (in our knowledge) using Transformers for UAV wildfire image segmentation. These models showed impressive results and outperformed recent published methods. They proved their ability in segmenting wildfire pixels, detecting the precise shape of fire. Based on the F1-score, TransFire obtained great performance, outperforming deep models such as EfficientSeg and U-Net. It also showed its excellent potential in detecting and segmenting forest fires and in overcoming challenging problems such as small fire areas and background complexity.

## Figures and Tables

**Table 1:** Deep learning methods for UAV-based fire classification.

Ref.	Methodology	Smoke/Flame	Dataset	Accuracy (%)
[34]	CNN-17	Flame/Smoke	Private dataset: 2100 images	86.00
[35]	AlexNet GoogLeNet Modified GoogLeNet VGG13 Modified VGG13	Flame	Private dataset: 23,053 images	94.80 99.00 96.90 86.20 96.20
[28]	Xception	Flame	FLAME dataset: 48,010 images	76.23
[36]	Fire_Net AlexNet	Flame	UAV_Fire dataset: 1540 images	98.00 97.10
[29]	VGG16 VGG19 ResNet50 InceptionV3 Xception	Flame	FLAME dataset: 8617 images	80.76 83.43 88.01 87.21 81.30
[37]	Fog computing and simple CNN	Flame	Private dataset: 2964 images	95.07
[38]	Fire_Net AlexNet MobileNetv2	Flame/Smoke	Private dataset: 2096 images	97.50 95.00 99.30

**Table 2:** Fire detection using Deep learning methods for UAVs.

Ref.	Methodology	Smoke/Flame	Dataset	Results (%)
[50]	YOLOv3	Flame	Private dataset: 3,840,000 images	F1-score = 81.0
[53]	YOLOv2 Faster R-CNN SSD	Smoke	Private dataset: 12,000 images	Accuracy = 98.3 Accuracy = 95.9 Accuracy = 81.1
[52]	YOLOv3	Flame/Smoke	Private dataset: 3,684,000 images	F1-score = 81.0
[57]	YOLOv3 and ARSB method	Flame	Private dataset: 1400 K images	mAP = 67.0

**Table 3:** Fire segmentation using deep learning methods for UAVs.

Ref.	Methodology	Smoke/Flame	Dataset	Results (%)
[58]	DeepLabV3+ DeepLabV3+ + validation approach	Flame/Smoke	Fire detection 360-degree dataset: 150 360-degree images	F1-score = 81.4 F1-score = 94.6
[60]	U-Net	Flame	FLAME dataset: 5137 images	F1-score = 87.7
[61]	U-Net CNN based on VGG16	Flame/Smoke	Private dataset: 366 images	Accuracy = 90.2 Accuracy = 93.4

**Table 4:** Dataset subsets for classification.

Dataset	Fire Images	Non-Fire Images
Training set	20,015	11,500
Validation set	5003	2875
Testing set	5137	3480

**Table 5:** Performance evaluation of wildfire classification models.

Models	Accuracy (%)	F1-Score (%)	Inference Time (s)
Xception	78.41	78.12	0.002
Xception [28]	76.23	—	—
EfficientNet-B5	75.82	73.90	0.010
EfficientNet-B4	69.93	65.51	0.008
EfficientNet-B3	65.81	64.02	0.004
EfficientNet-B2	66.04	60.71	0.002
InceptionV3	80.88	79.53	0.002
DenseNet169	80.62	79.40	0.003
MobileNetV3-Small	51.64	44.97	**0.001**
MobileNetV3-Large	65.10	60.91	**0.001**
**Proposed ensemble model**	**85.12**	**84.77**	0.018

**Table 6:** Performance evaluation of wildfire segmentation models.

Models	Accuracy (%)	F1-Score (%)	Inference Time (s)
TransUNet-R50-ViT	**99.90**	**99.90**	0.51
TransUNet-ViT	99.86	99.86	0.40
TransFire	99.83	99.82	1.00
EfficientSeg	99.63	99.66	1.38
U-Net	99.00	99.00	**0.29**

## Data Availability

This work uses a publicly FLAME dataset, which is available on IEEE-Dataport [45]. More details about the data are available under Section 3.3.
